# Endoscopic submucosal dissection for pharyngeal cancer: removal of supra-oropharyngeal cancer

**DOI:** 10.1016/j.vgie.2023.03.004

**Published:** 2023-05-05

**Authors:** Ryogo Minami, Ayu Tachibana, Yoshiaki Moriguchi, Eriko Noma, Takeo Arakawa, Tomoko Oonishi, Shinichiro Horiguchi, Toshiro Iizuka

**Affiliations:** 1 Department of Gastroenterology, Tokyo Metropolitan Cancer and Infectious Diseases Center, Komagome Hospital, Tokyo, Japan; 2 Department of Pathology, Tokyo Metropolitan Cancer and Infectious Diseases Center, Komagome Hospital, Tokyo, Japan; 3 Department of Gastroenterology, Tokyo Metropolitan Cancer and Infectious Diseases Center, Komagome Hospital, Tokyo, Japan

## Abstract

Video 1

## Introduction

Endoscopic submucosal dissection (ESD) for superficial pharyngeal carcinomas is a useful, minimally invasive treatment.^1^^,^^2^ However, resecting a lesion, especially one in the oral cavity where the surgical space is limited and complicated, is technically challenging. Herein we reported ESD in the treatment of supra-oropharyngeal cancer, which we performed with the patient under general anesthesia induced by nasotracheal intubation.

## Case

The patient was a 77-year-old man with a history of esophageal and pharyngeal ESD for a superficial squamous cell carcinoma. A follow-up endoscopy revealed melanosis in the right palatine arch. A biopsy of the site revealed squamous cell carcinoma in situ with melanosis. The previous pharyngeal ESD was performed for left pyriform sinus (20 × 12 mm, carcinoma in situ, ly0, v0, pHM0, pVM0). Therefore, it is unlikely to be a recurrence. A CT scan showed no evidence of metastasis.

## Procedure

**Figure 1:**
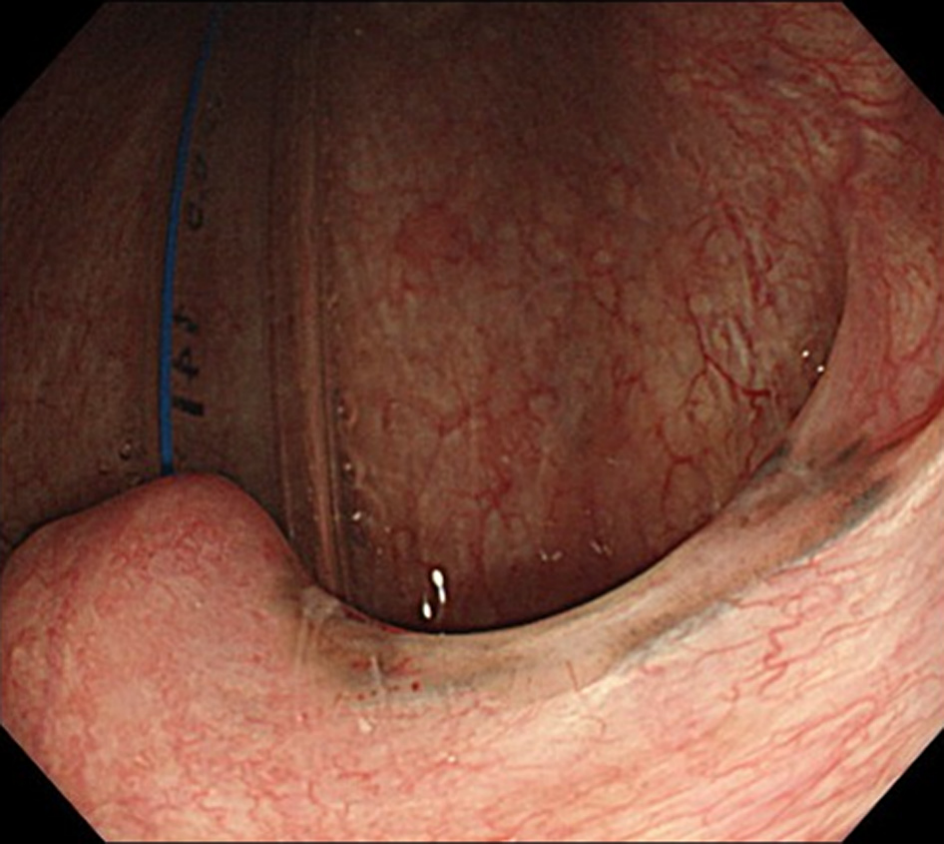
Endoscopic image before retroflexion. Melanosis was seen on the right palatine arch.

**Figure 2:**
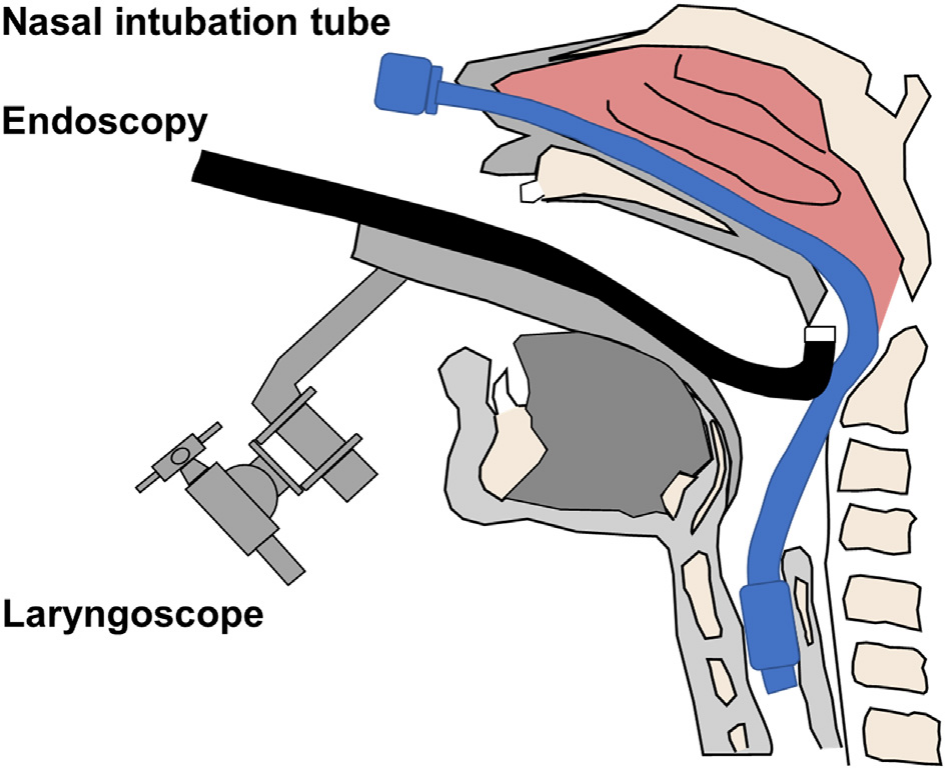
Schematic view of the surgical setup. The tip of the laryngoscope was placed in front of the vocal cords.

**Figure 3:**
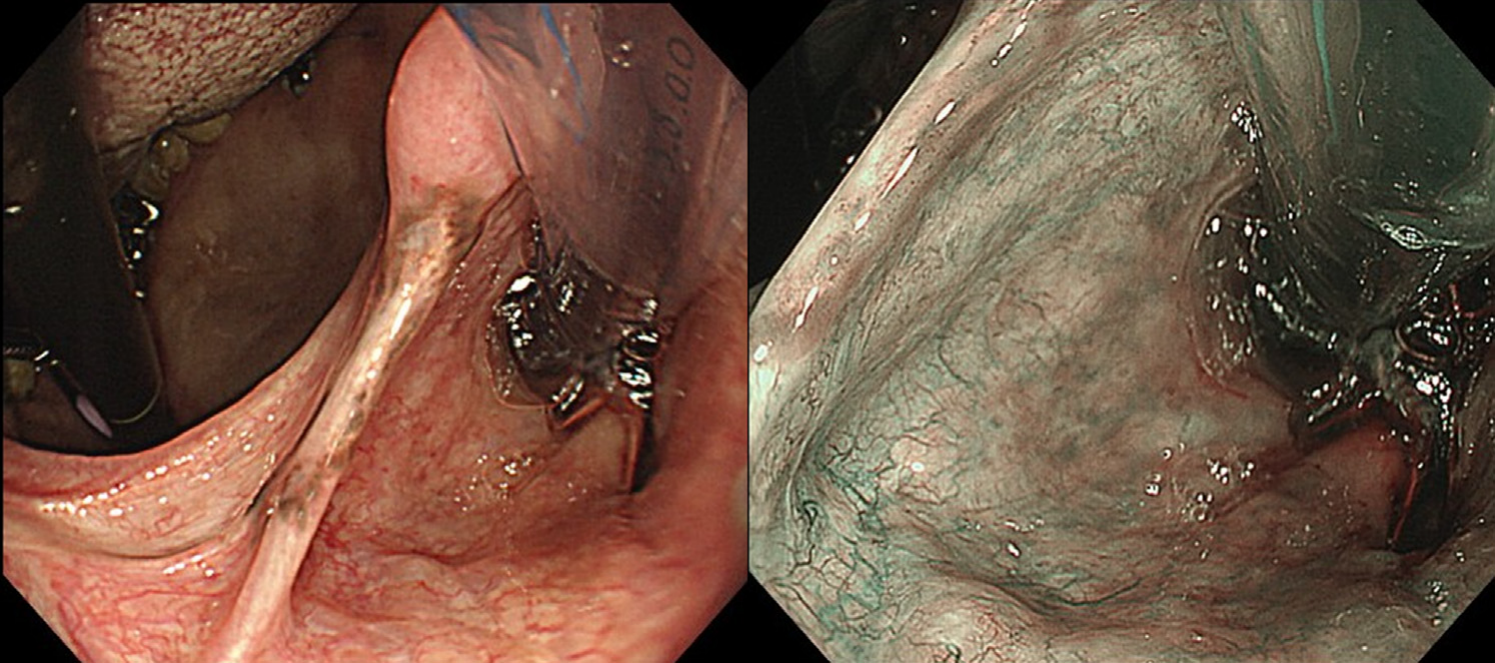
Endoscopic image during retroflexion. The lesion showed melanosis and slight extension to the supra-pharyngeal area.

**Figure 4:**
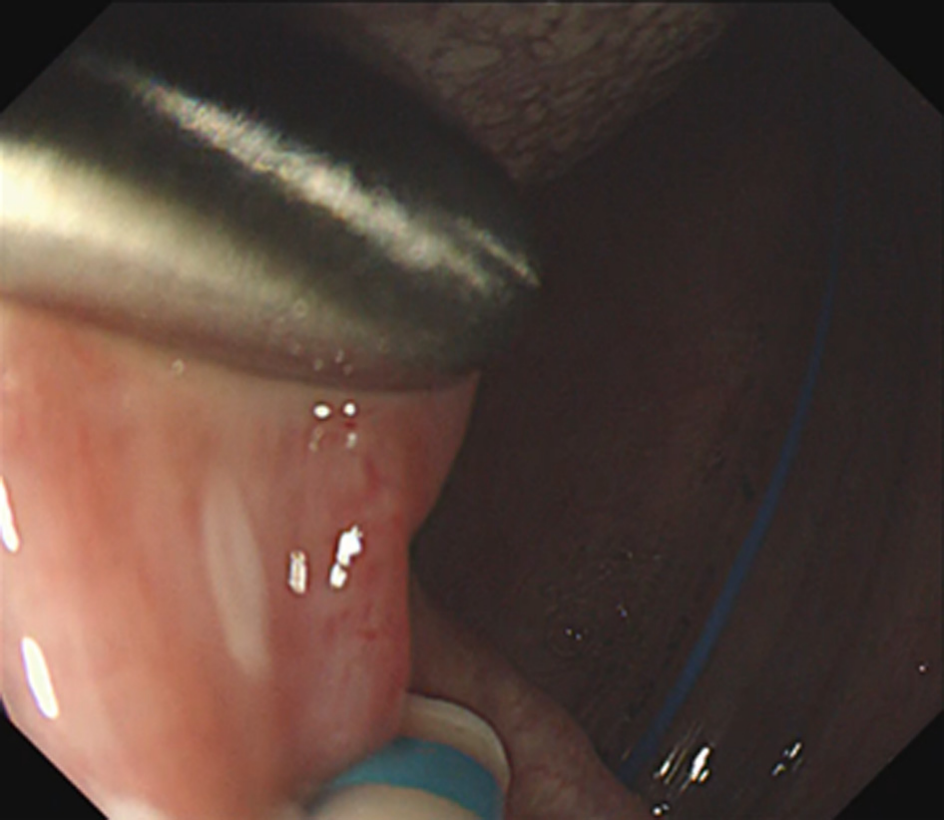
Endoscopic image of the marking process. The uvula was grasped using forceps better to visualize the boundaries of the supra-pharyngeal site.

**Figure 5:**
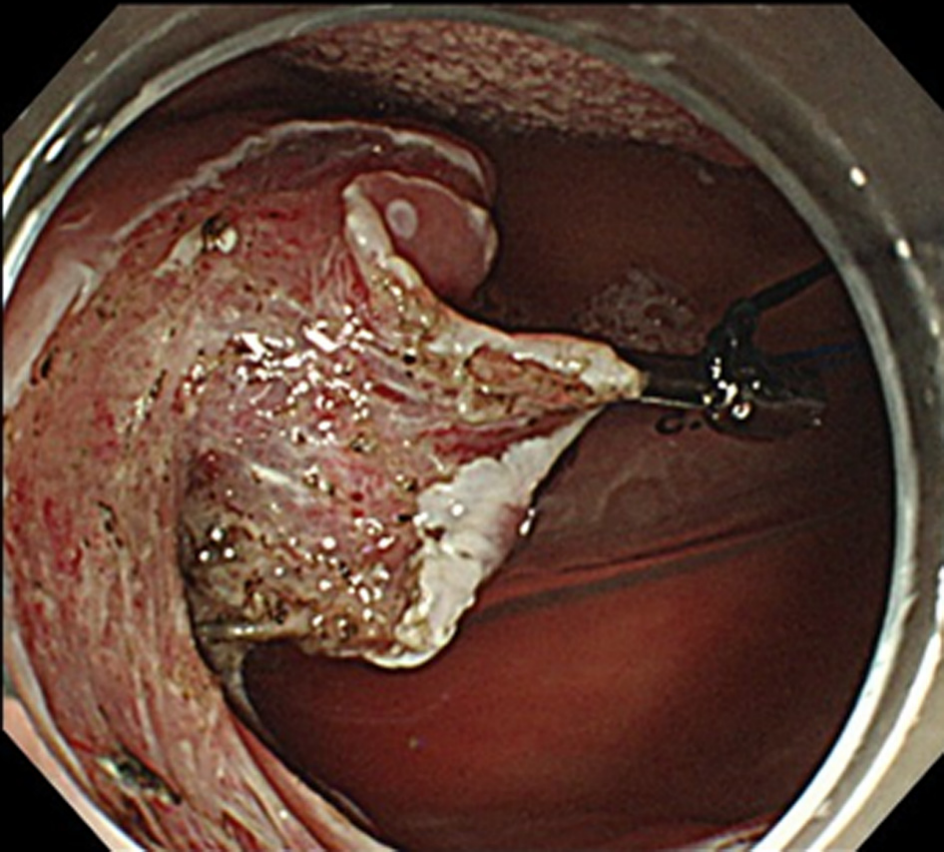
Endoscopic image during dissection. A clip with a line was used to apply traction.

**Figure 6:**
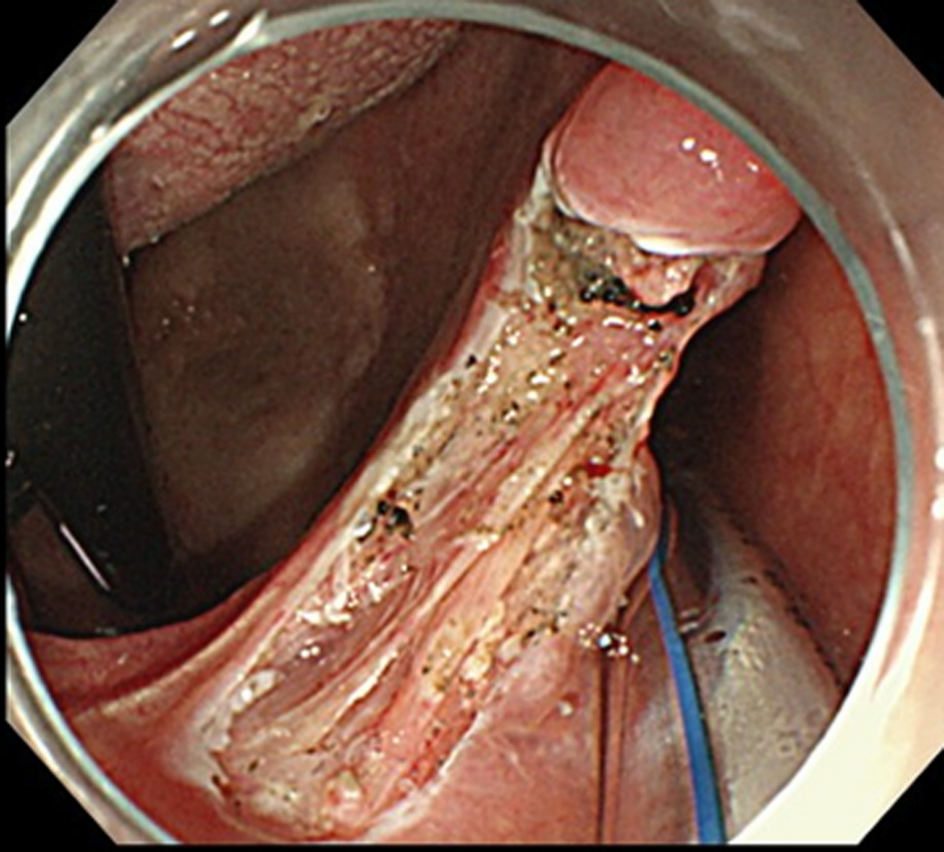
Endoscopic submucosal dissection ulcer after resection. The lesion was resected without adverse events.

ESD was performed in the operating room while the patient was under general anesthesia induced via nasal intubation (Fig. 1). The tip of a curved laryngoscope (Nagashima Medical Instruments Co, Ltd, Tokyo, Japan) was placed in front of the vocal cords, and the lesion was observed using GIF-H260Z (Olympus, Tokyo, Japan). Retroflexion was used to insert the endoscope to visualize the nasopharyngeal margins more clearly (Fig. 2). The lesion on the right palatine arch demonstrated melanosis and extension toward the supra-pharyngeal area (Fig. 3). The tumor type was macroscopically identified as type 0-Ⅱb.A needle-type ESD knife (Dual Knife; Olympus) was used to mark dots around the circumference of the lesion in preparation for a circumferential incision and dissection (Fig. 4). To dissect the subepithelial layer on the nasopharyngeal side, a clip with line was used (Fig. 5). Dissection was straightforwardly carried out, and the lesion was resected en bloc without any adverse events (Fig. 6; Video 1, available online at www.videogie.org).

**Figure 7:**
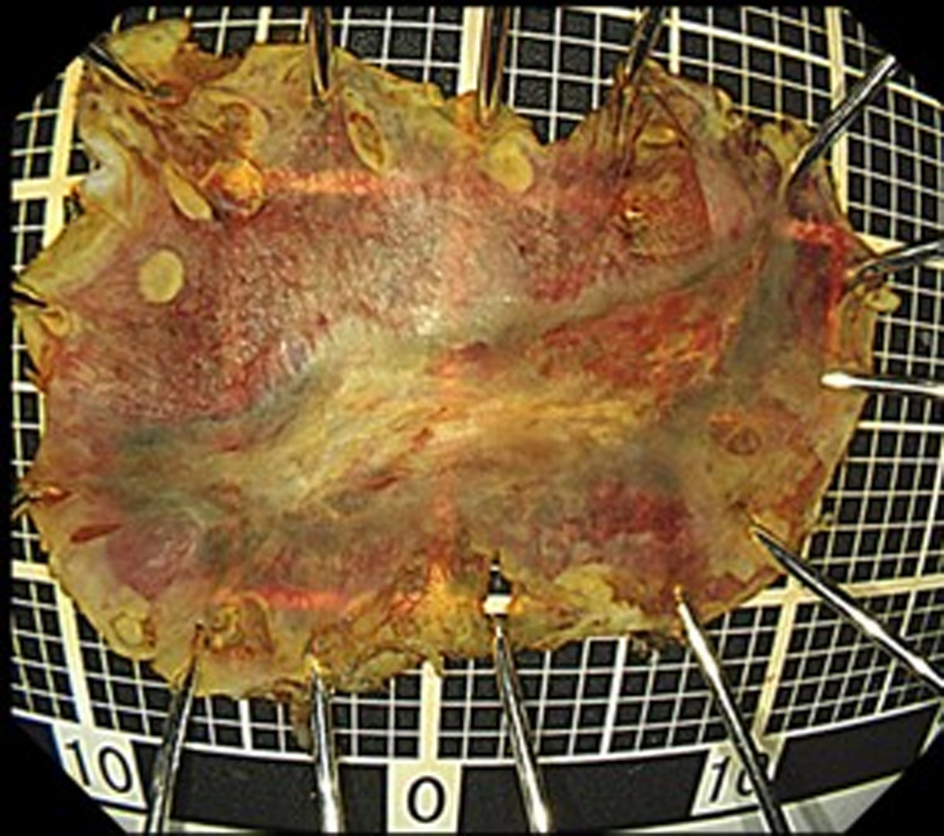
Endoscopic submucosal dissection specimen. Iodine staining for the specimen suggested complete resection.

**Figure 8:**
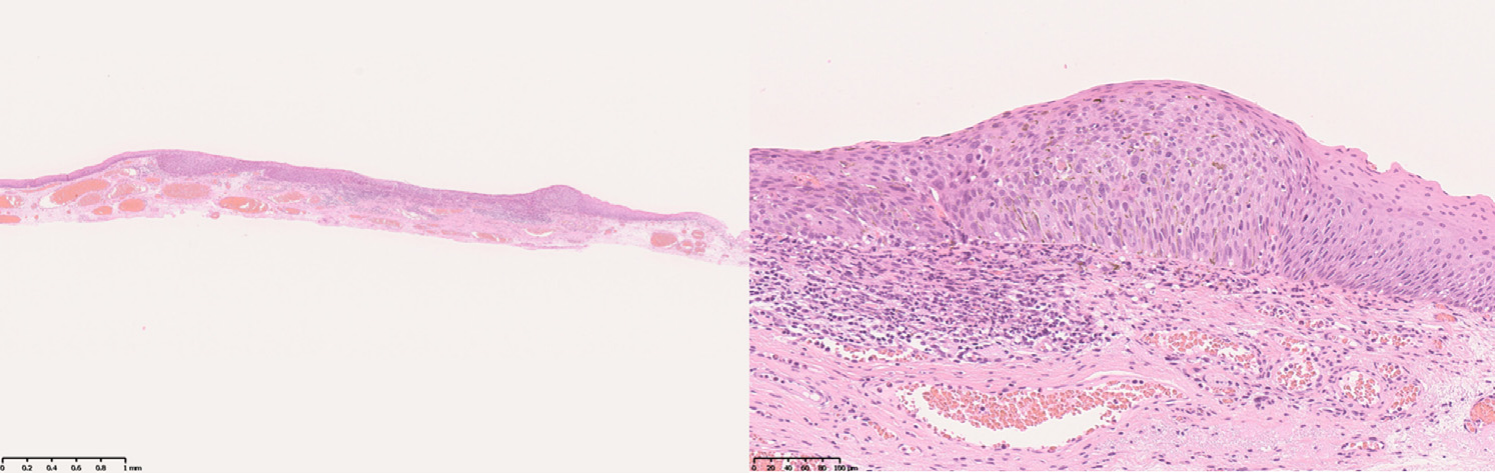
Histopathologic image of the resected specimen. The lesion was a squamous cell carcinoma in situ.

**Figure 9:**
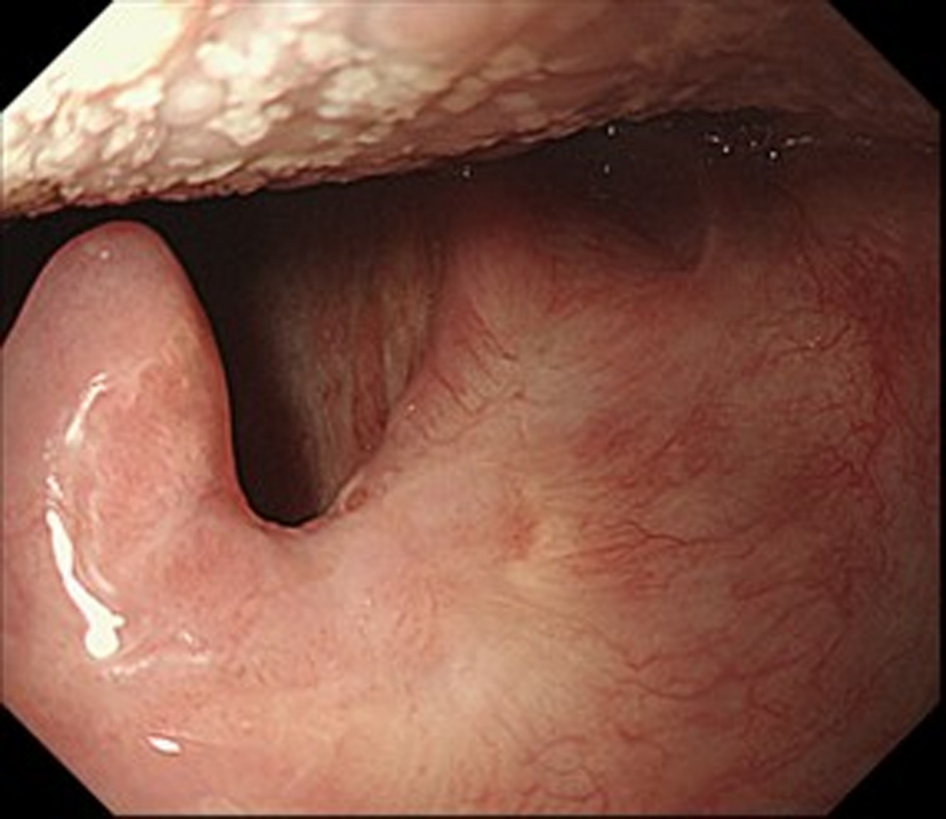
One month after endoscopic submucosal dissection. The surgical wound has completely healed.

The duration from marking the circumference of the lesion to dissection was 31 minutes. The resected specimen measured 22 × 16 mm (Fig. 7). Histopathological analysis revealed squamous cell carcinoma with a tumor thickness of 280 μm and negative horizontal and vertical margins. No lymphovascular invasion was detected (Fig. 8). In the post-ESD course, the patient’s pain was within his control and he did not want to use analgesics. Food intake was resumed on day 2 post-ESD, and the patient was discharged on day 7 post-ESD. At 1-month post-ESD, a follow-up endoscopy revealed that the surgical wound had completely healed (Fig. 9).

## Discussion

To the best of our knowledge, the present study is the first to report ESD performed for a pharyngeal carcinoma extending to the supra-pharynx. In almost all previous reports of superficial pharyngeal cancer, the lesion occurred in the pyriform sinus or the posterior or lateral pharyngeal wall or the uvula.^3^^,^^4^ ESD for an oral lesion is challenging because of the difficulty of obtaining an unobstructed field of view (working space) owing to the presence of the tongue and narrow lumen. In the present case, several distinctive innovations were used to perform the ESD. First, nasal intubation was performed without the intubation tube interfering with the movements of the endoscope. Second, observation during retroflexion was used to determine the extent of the lesion. Owing to the limited range of motion in retroflexion, the endoscope was used only for observation while in this position. The clip with a line was crucial for dissecting the nasopharyngeal side of the lesion. Applying traction with forceps reduced the time required for dissection. Third, elevating the larynx using the curved laryngoscope facilitated securing the working space.

Superficial oropharyngeal cancer is difficult to detect using an otolaryngology endoscope, which is incapable of magnification. However, it is often detected by gastroenterologists using a magnifying endoscope. Whether superficial oropharyngeal cancer is treated in the otolaryngological or gastroenterological department depends on the institution. At our hospital, the department where the lesion is first detected often undertakes the treatment. We perform an ESD for oropharyngeal cancer, regardless of size, if it is superficial. If the lesion extends into the nasopharynx, it is considered treatable as long as it can be approached using the inversion maneuver.

Our case demonstrated that ESD for pharyngeal cancer on the palatopharyngeal arch extending into the nasopharynx may be feasible if nasal intubation and a curved laryngoscope are used.

## Disclosure

*The authors disclosed no financial relationships.*
